# Reviews, Notices, &c.

**Published:** 1855-12

**Authors:** 


### REVIEWS, NOTICES, &C.

*Medical Pronouncing Lexicon,* by Prof. C. H. Cleaveland, M. D., of Cincinnati, 302 pp. 16 mo. A publication like this is essential to every medical student, and should be made a pocket companion for hourly reference, at least until graduation. The classical scholar will remember that Virgil suffered greatly in his reputation from the mispronunciation of a single word; and to pronounce a medical term incorrectly now, instantly lowers the character for scholarship in the estimation of. an intelligent hearer, and it will require long subsequent acquaintance for replacement. Prof. C. has discharged his duty ably by means of the American Phonetic Alphabet. But the correctness and nicety of pronunciation are attained by the addition of some forty new characters which we consider an insuperable objection. The requirements of the age are to simplify. We need fewer letters in the alphabet than we already have, instead of twenty more capitals, and as many more of their representative smalls. Still we must confess, that for accent and pronunciation it is more concise, more compact, than the explanations for the same purpose at the bottom of the pages of ordinary dictionaries, and for correctness of definition is invaluable.

---

*Littell's Living Age,* weekly, 62 pp. 8 vo., double column, at $6 a year. Among other valuable articles in No. 600 are, The First Edinburgh Reviewers; Peter the Great in England; Half a Life Time Ago; Women in Turkey; England's Danger and Discredit, &c. &c.

---

*Boston Medical and Surgical Journal,* for December, has several sterling articles on medical literature.

